# Age-Dependent Estimates of the Epidemiological Impact of Pandemic Influenza (H1N1-2009) in Japan

**DOI:** 10.1155/2013/637064

**Published:** 2013-02-21

**Authors:** Kenji Mizumoto, Taro Yamamoto, Hiroshi Nishiura

**Affiliations:** ^1^School of Public Health, The University of Hong Kong, Level 6, Core F, Cyberport 3, 100 Cyberport Road, Pokfulam, Hong Kong; ^2^Department of International Health, Nagasaki University Institute of Tropical Medicine and GCOE, Sakamoto, Nagasaki 852-8523, Japan; ^3^PRESTO, Japan Science and Technology Agency, Saitama 332-0012, Japan

## Abstract

The total number of influenza cases with medical attendance has been estimated from sentinel surveillance data in Japan under a random sampling assumption of sentinel medical institutions among the total medical institutions. The 2009 pandemic offered a research opportunity to validate the sentinel-based estimation method using the estimated proportion of infections measured by the population-wide seroepidemiological survey employing hemagglutinin inhibition (HI) assay. For the entire population, we estimated the age-standardized proportion of infections at 28.5% and 23.5% using cut-off values of HI titer at 1 : 20 and 1 : 40, respectively. Investigating the age profiles, we show that the estimated influenza-like illness (ILI) cases with medical attendance exceeded the estimated infections among those aged from 0 to 19 years, indicating an overestimation of the magnitude by sentinel-based estimation method. The ratio of estimated cases to estimated infections decreased as a function of age. Examining the geographic distributions, no positive correlation was identified between the estimated cases and infections. Our findings indicate a serious technical limitation of the so-called multiplier method in appropriately quantifying the risk of influenza due to limited specificity of ILI and reporting bias. A seroepidemiological study should be planned in advance of a pandemic.

## 1. Introduction

To implement effective infectious disease control, it is vital that the information of any epidemic is continuously and systematically collected, and the results of data analysis should be effectively shared with the public and experts in a timely manner [1]. Several different types of epidemiological surveillance have been put in place in Japan and played important roles in measuring the epidemiological impact of the pandemic influenza A (H1N1-2009), including those allowing the estimation of the total numbers of infections, severe cases, and deaths [2, 3]. Among these indices, it is useful to measure the cumulative incidence of infection in order to estimate the extent of infection (or the risk of infection) throughout the course of the pandemic, to understand the overall population impact of infections (e.g., hospital burden given an estimated number of infections), and to estimate the severity of infection at an individual level using additional data of severe cases or deaths. As an important lesson from the 2009 pandemic, the use of serological data, for example, the population-wide cross-sectional survey that employs hemagglutination inhibition (HI) assay, appears to be useful for directly measuring the proportion of those who experienced infection [4–6].

Spanning decades, a sentinel surveillance system has been established as a part of routine surveillance practice in Japan. Using the sentinel data, Japan has adopted the so-called multiplier method to estimate the total number of clinically apparent influenza cases with medical attendance [7], the methodological details of which are described below in this paper (see Section 2). The estimation of the total number of cases was not the primary objective of the sentinel surveillance, and rather, continuous epidemiological monitoring and early detection of outbreaks have been in line with the main scope of the sentinel surveillance system [8]. However, the estimates of influenza cases based on the multiplier method have been routinely obtained and announced to the public, adopting a random sampling assumption of sentinel medical institutions among the total medical institutions. While knowing that the estimates are very crude among experts, it has been frequently the case that the given numbers are only available estimates of cases across Japan, and the “estimated cases” have been adopted as if they were officially accepted number of clinically apparent cases through mass media. Such estimates have been used for assessing the severity of influenza (H1N1-2009) not only in Japan but also in other countries [9–11]. Moreover, the estimates have been conventionally used for a variety of epidemiological studies (e.g., a study that characterizes the descriptive epidemiology of severe cases of the 2009 pandemic [12]).

When it comes to the seroepidemiological assessment by using HI assay, there are several advantages for the 2009 influenza pandemic to directly measure the extent of infection by using the serological testing. First, the majority of the population has been naive to the influenza A (H1N1-2009) and the virus has been shown not to yield cross-reaction with past influenza viruses except among the elderly. Second, the time length of the first wave from 2009 to 2010 was evident in the Northern hemisphere, and thus, it was possible to determine two time points to collect blood samples (i.e., before and after the first wave) without ambiguity. Third, vaccination coverage remained low during the first wave, and thus, the serological data after the pandemic was less influenced by vaccination than after seasonal influenza epidemics (i.e., the seropositivity reflects naturally occurring infections). Namely, the 2009 pandemic offers an important opportunity to directly measure the cumulative incidence by means of seroepidemiological survey, thereby allowing us to critically assess the estimated number of ILI cases based on sentinel surveillance data.

The present study aims to estimate and compare the age-specific cumulative incidence of infections and clinical cases with medical attendance during the first wave of the 2009 pandemic based on different sources of data. In particular, we focus on the age profile and the geographic pattern.

## 2. Materials and Methods

### 2.1. Data Source

In the present study, we used three different pieces of information, that is, (a) the seroepidemiological survey data, (b) the estimated number of cases from sentinel surveillance system, and (c) the estimated risk of infection based on a mathematical model that was parameterized by using the confirmed case data by the end of July 2009. First, the seroepidemiological data were derived from the National Epidemiological Surveillance of Vaccine-Preventable Diseases (NESVPD) [13]. In Japan, the immunity profile at a population level has been routinely measured for eight of the selected vaccine-preventable infectious diseases that include influenza. The survey has taken place annually, collecting serum from at least 5,400 nonrandomly sampled individuals across all age groups based on area sampling. The voluntary participants were manually recruited at prefectural levels, frequently among those who visited the prefectural hospital or public health center for medical purposes other than antibody testing. During the serum collection, the past medical history (including influenza) and vaccination history were also collected. Geographically, the sampling took place in 23 prefectures with 225 individuals per prefecture, among a total of 49 prefectures. In both 2009 and 2010, the serum samples were obtained from July to September. In 2009, the sampling time corresponded to the beginning of the first wave [14], while in 2010 the time of sampling was between the end of the first wave and the time to start vaccination for the next influenza season. HI antibody titer was measured and summarized by age and prefecture.

Second, the notification of influenza was made from the sentinel medical institutions that consist of 4,800 randomly sampled hospitals or clinics in Japan. Physicians at the sentinel medical institutions were obliged to report the number of influenza-like illness (ILI) cases to the government, and the cases should meet the following criteria: (a) acute course of illness (i.e., sudden onset), (b) fever greater than 38.0°C, (c) cough, sputum, or breathlessness (upper respiratory tract infection symptoms), and (d) general fatigue, or who were strongly suspected of the disease by undertaking laboratory diagnosis (e.g., rapid diagnostic testing). Using the reported number of ILI cases and the proportion of sentinel institutions among the total medical institutions (which is approximately 10% during the 2009 pandemic), the estimated total number of ILI cases was calculated (see Section 2.2). Assuming that the sentinel medical institutions were randomly recruited from all medical institutions in Japan, the estimated number of influenza cases would represent “all clinically ill influenza cases who sought medical attendance” (and does not include asymptomatic cases and mild symptomatic cases without medical attendance) [15]. To assess the epidemiological impact of the 2009 pandemic, we investigated the estimated number of cases from week 28 in 2009 (i.e., the week ending on July 12 2009) to week 10 in 2010 (i.e., the week ending on March 14 2010) which is consistent with the length of time for the first wave.

Third, a mathematical modeling study was conducted after the containment phase in Japan, which was discontinued at the end of July 2009, analyzing the age-dependent transmission dynamics and quantifying the age-dependent next generation matrix [14]. Using the next generation matrix, the age-dependent cumulative risk of infection (i.e., the final size) was analytically computed. To assess the qualitative validity of the model-based prediction after the containment phase, the predicted age-dependent risk was used as supplement to two other estimates in the present study.

### 2.2. Statistical Estimation

From the two sets of seroepidemiological surveys, we have obtained two distributions of HI titer by age group. The sampling of two surveys was not made from the same individuals, and thus, we cannot unfortunately take the ratio of the titer at an individual level. Thus, we adopted two different cut-off values to define seropositivity, that is, one using the conventional antibody titers ≥1 : 40 by HI assay and the other ≥1 : 20 intending the latter to be a part of sensitivity analysis. Accordingly, we obtained the seroprevalence before and after the first wave, *p*_1_ and *p*_2_, respectively, and we calculated the cumulative incidence of infection during the first wave, *q*, as *q* = *p*_2_ − *p*_1_. Let the sample sizes of two surveys be *n*_1_ and *n*_2_, respectively, the standard error of the cumulative incidence *q* under an independence assumption is calculated as

(1) $$\begin{array}{c} s.e.\left(q\right)=\sqrt{\frac{p_{1}\left(1-p_{1}\right)}{n_{1}}+\frac{p_{2}\left(1-p_{2}\right)}{n_{2}}}. \end{array}$$

Since the population size of Japan is sufficiently large, we ignored the demographic stochasticity of the major epidemic (as was discussed elsewhere [16]) and assumed that the distribution of the risk of infection is sufficiently approximated by a binomial distribution. Subsequently, the 95% confidence interval of the cumulative incidence was calculated as *q* ± 1.96 × *s*.*e*.(*q*).

As for the sentinel-based estimation, we used the estimated number of cases with medical attendance that had been calculated as follows. First, suppose that there were *m* medical institutions in Japan among which *M* were selected as sentinel medical institutions. Let *i* be the notified number of cases from a sentinel medical institution in a given reporting interval, and let *M*_*i*_ be the number of sentinel medical institutions with *i* notifications of influenza. Assuming that the sentinel medical institutions *M* are the random samples of all medical institutions *m* with a probability (*M*/*m*), the total number of cases, *k*, during the corresponding time interval has been calculated as

(2) $$\begin{array}{c} k=\frac{m}{M}\sum_{i}iM_{i}. \end{array}$$

Because of the critical role of *m*/*M* in scaling the estimated number of clinical cases, the estimation method using (2) (or something similar) is referred to as the multiplier method. The description of the computation of approximate confidence interval is given elsewhere [7]. In the present study, we used only the expected values by age group, because they were only the obtainable data. The notifications from each sentinel medical institution, *M*_*i*_ were not openly accessible.

From the age-dependent epidemiological model, we used the next generation matrix {*R*_*ij*_} that was quantified elsewhere [14]. The predicted final size (or the cumulative incidence of infection) *z*_*i*_ for age group *i* was computed by solving the following final size equation [14]:

(3) $$\begin{array}{c} z_{i}=1-\sum_{j}z_{j}R_{ij}. \end{array}$$

Since the age bands were different by different datasets, and because the uncertainty bounds (e.g., the 95% confidence intervals (CI)) were partly not accessible, we did not employ an explicit hypothesis testing method to compare the estimates based on two or more methods. At least, we computed the 95% CI, where possible, and overlaid the expected values of the age-dependent estimates of infections and cases to ease the comparison. In addition, the median and quartiles were computed as representing the descriptive data of the skewed distributions of infections and cases. Wherever possible, we examined the age-dependent estimates of infections and clinical cases in addition to crude estimates. Since the ILI data by prefecture were not age stratified, we compared it against the standardized cumulative incidence of infection based on serological data weighted by age-specific population size of the entire Japan. Moreover, we calculated the age-dependent ratio of ILI cases to the cumulative incidence of infection in order to understand the age specificity of the bias in the case estimate. All statistical data were analyzed using a statistical software JMP version 9.0.0 (SAS Institute Inc., Cary, NC, USA).

## 3. Results

### 3.1. Distributions of the HI Antibody Titer in 2009 and 2010

**Figure 1:**
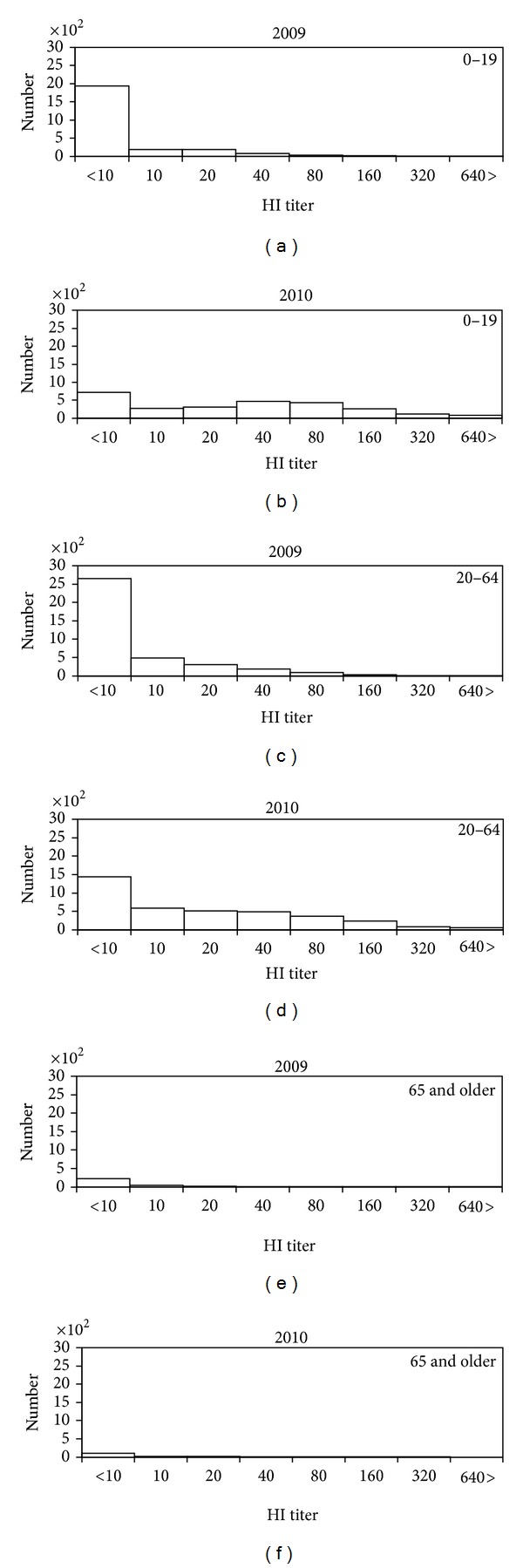
Distribution of the antibody titer of hemagglutination inhibition (HI) assay in 2009 and 2010. ((a), (c), and (e)) 2009 and ((b), (d), and (f)) 2010. ((a) and (b)) 0–19 years old. ((c) and (d)) 20–64 years old. ((e) and (f)) 65 years old and older. In both years, the survey took place from July to September.

Figure 1 compares the distributions of the HI antibody titer between 2009 and 2010. The mean and median HI titers among those aged from 0–19 years were 17.2 and 5.0 (quartile: 5.0, 5.0), respectively, in 2009, while they were 119.3 and 60.0 (quartile: 5.0, 120.0), respectively, in 2010. Among those aged from 20 to 64 years, the mean and median HI titers in 2009 were 18.9 and 5.0 (quartile: 5.0, 15.0), respectively, whereas they were 72.4 and 15.0 (quartile: 5.0, 60.0), respectively, in 2010. The increase among the elderly (those aged 65 years or older) was subtle; the mean titer was elevated from 24.6 in 2009 to 33.0 in 2010. According to personal recall among 3876 participants, the vaccination against H1N1-2009 allegedly took place in 1402 individuals. Thus, the vaccination coverage was 36.2% (95% confidence interval: 34.7, 37.7) which was mostly seen in the elderly due to prioritized real-time vaccination during the course of the pandemic.

### 3.2. Influenza Cases with Medical Attendance

**Figure 2:**
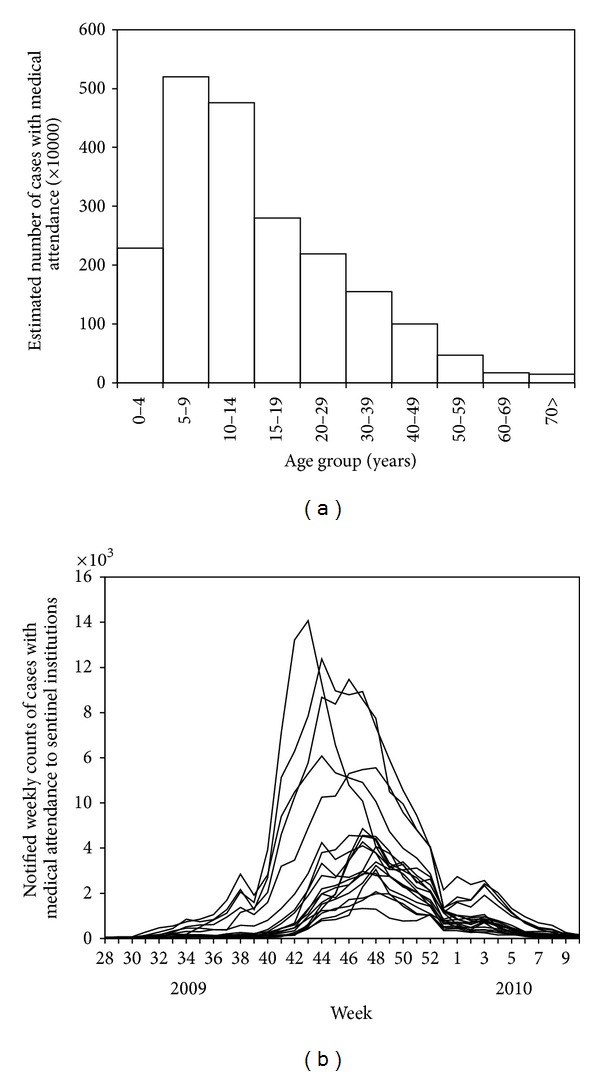
Influenza-like illness cases with medical attendance in Japan from 2009 to 2010. (a) The estimated number of influenza-like illness (ILI) cases with medical attendance is given for the period from week 28 in 2009 to week 10 in 2010, corresponding to the first wave of the 2009 pandemic. The estimated number was obtained from the reported number of ILI cases with medical attendance and the proportion of sentinel medical institutions among the total number of medical institutions across Japan. (b) The notified weekly number of influenza cases with medical attendance by prefecture. The datasets from a total of 19 prefectures in which the serological survey took place are shown.

Figure 2(a) shows the estimated number of influenza cases with medical attendance for the period from week 28 in 2009 to week 10 in 2010. The distribution appeared to be right-skewed with a mode among those aged from 5 to 9 years with the estimated age-specific number of 520 × 10^4^ cases across Japan. The mean and median ages of cases were 17.3 and 12.5 (quartile: 7.5, 25.0) years, respectively. For the entire Japan, the estimated total number of cases with medical attendance during the corresponding period was 2,066 × 10^4^ cases.  Figure 2(b) illustrates the notified weekly counts of influenza cases with medical attendance by prefecture (*n* = 19). The largest notifications (i.e., the peaks of epidemic curve) were seen from week 43 to 49 in 2009. There was a tendency that the epidemic wave peaks and falls off in about 30 weeks.

### 3.3. Estimated Cumulative Incidence of Infections and Cases

**Figure 3:**
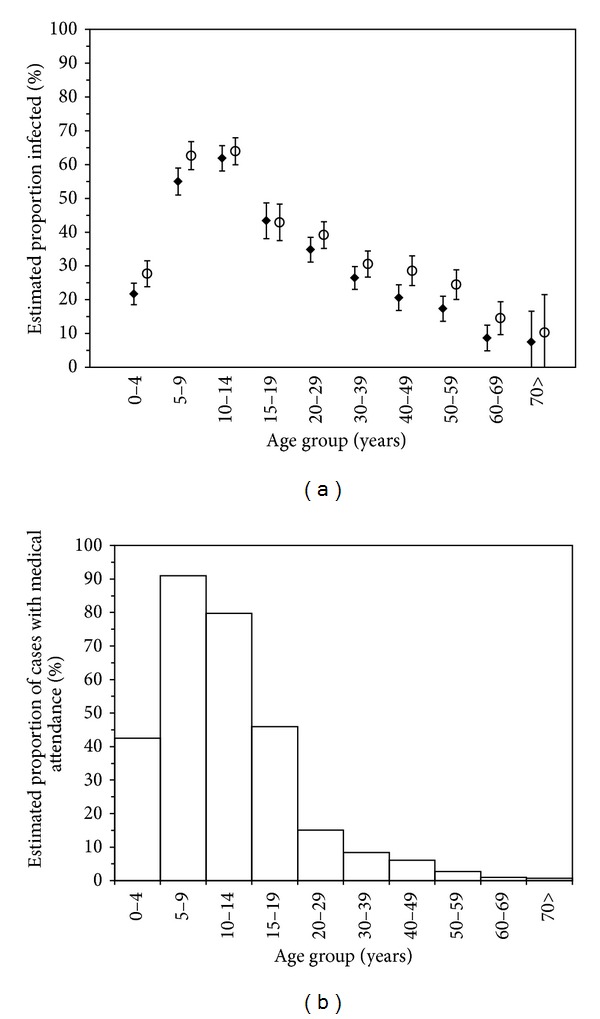
Estimated proportions of influenza infections and cases from 2009 to 2010. (a) The estimated proportion infected by age group. Ignoring short-term decay in antibody titer, the proportion infected during the first wave of the 2009 pandemic was calculated as the difference between the proportions above a cut-off level of titer in 2009 and 2010. In both years, the survey took place from July to September. We adopted 1 : 20 (unfilled circles) and 1 : 40 (filled diamonds) as the cut-off values. Whiskers extend from lower to upper 95% confidence intervals. (b) The estimated proportion of influenza-like illness (ILI) cases with medical attendance by age group. The notified number of cases from week 28 in 2009 to week 10 in 2010 was used to estimate the total number of ILI cases with medical attendance, using the proportion of sentinel medical institutions among the total number of medical institutions across Japan.

Figure 3(a) shows the estimated cumulative incidence of infections based on two serological surveys. The distributions using 1 : 20 and 1 : 40 did not yield significantly different estimates, and the mean and median ages of infection were 24.7 and 17.5 years (quartile: 7.5, 35.0), respectively, using the cut-off value of 1 : 20; 22.8 and 17.5 years (quartile: 7.5, 35.0), respectively, using the cut-off value of 1 : 40. The highest estimates were seen among those aged from 10 to 14 years, and the estimated cumulative incidence of this age group was 64.0% (95% CI: 60.0, 67.9) for 1 : 20 and 61.8% (95% CI: 58.1, 65.6) for 1 : 40, respectively. Age-standardized cumulative incidence for the entire Japan was 28.5% and 23.5% for 1 : 20 and 1 : 40, respectively, although it should be noted that the estimates were slightly overestimated due to vaccination.  Figure 3(b) shows the estimated cases with medical attendance across Japan, expressed as the cumulative incidence of ILI cases among the total population. The peak was seen among those aged from 5 to 9 years with the estimated cumulative incidence of 90.9% followed by those aged from 10 to 14 with 79.7%. The crude estimate of the cumulative incidence of cases with medical attendance was 16.1%.

### 3.4. Comparison: Age Profile and Geographic Distribution

**Figure 4:**
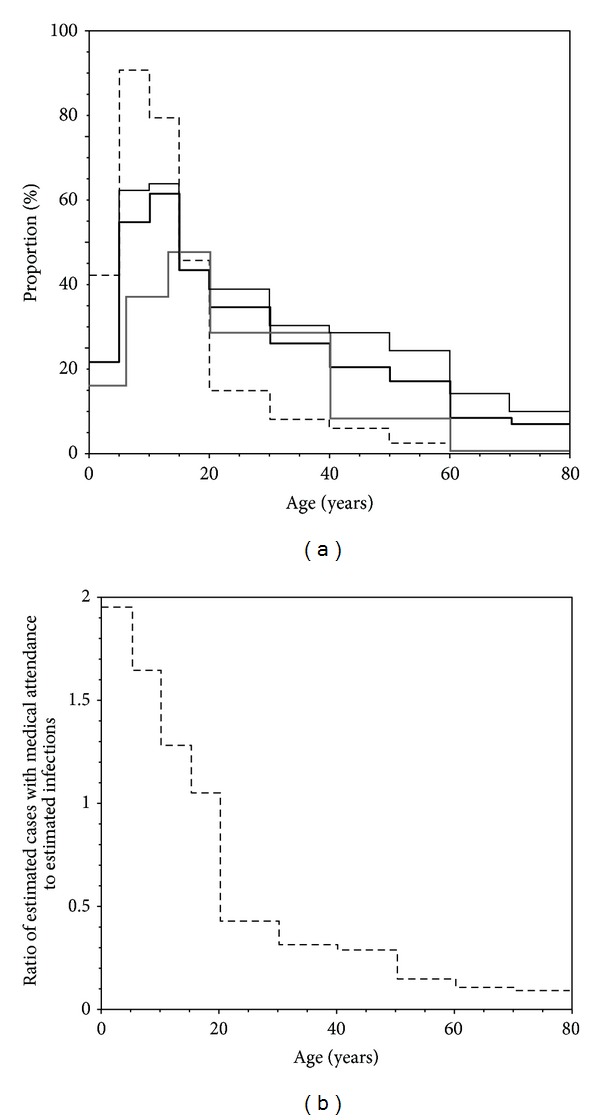
Comparison of the estimated proportions of infection with pandemic influenza H1N1-2009 in Japan using different methods. (a) Comparison of four different datasets. Dashed line represents the estimated influenza-like illness (ILI) cases with medical attendance based on notified number of ILI cases from sentinel medical institutions. Bold continuous line is the baseline proportion of infections based on hemagglutination inhibition assay adopting a cut-off value of 1 : 40. Thin continuous line represents the alternative serological measure of the proportion infected using a cut-off value of 1 : 20. Grey bold line shows the predicted cumulative incidence derived from the final size equation of an age-structured mathematical model based on the datasets by the end of July 2009. (b) The ratio of estimated cases with medical attendance based on notification data to the estimated proportion infected individuals based on serological data as a function of age group.

Figure 4(a) compares four different estimates: (i and ii) the estimated cumulative incidence of infection based on serological data (with two different cut-off values), (iii) the estimated cumulative incidence of infection calculated from the final size equation, and (iv) the estimated cumulative incidence of ILI cases based on sentinel surveillance. Overall, all four lines exhibited a similar qualitative age-specific pattern in which the estimate is highest among children and tapers as people becomes older. A remarkable sharp peak among those aged from 5 to 9 years was observed for the estimated ILI cases with medical attendance, which even yielded greater estimates than the estimated “infection” by seroepidemiological survey and mathematical model. In addition to 5–9-year-old group, the estimated ILI cases with medical attendance exceeded serologically estimated proportion of infections among those aged 0–4, 10–14, and 15–19 years, respectively. Among adults, the estimated cases were below the estimated infections. Compared to serological estimates of infection, the mathematical model underestimated the cumulative incidence among those aged below 12 years old, although the model-based estimates in other age groups were qualitatively and crudely in agreement with the serological estimates.

Figure 4(b) shows the ratio of estimated cases with medical attendance based on sentinel surveillance to estimated infections based on serological data as a function of age group. The ratio appeared not to be one (or a constant, that is, it should ideally be a constant below unity), even approximately, across all age groups. The overestimation of ILI cases would more likely be made among children as compared to adults. The calculated ratio among the youngest age group (at the age of 0–4 years) was close to 2.0, while the ratio among the oldest elderly was close to 0.1.

**Figure 5:**
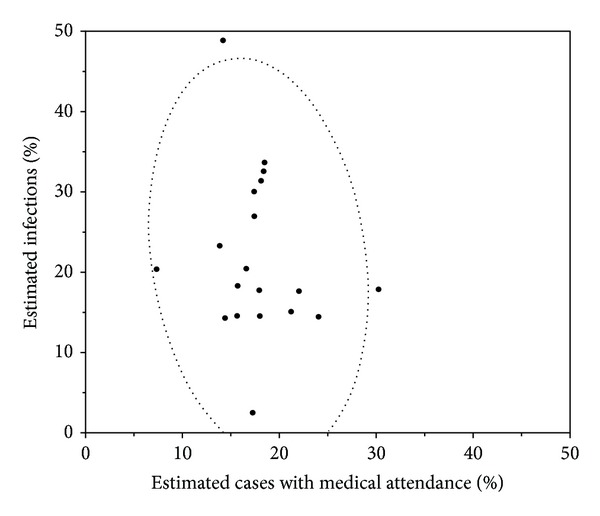
Estimated proportion infected and the estimated cases with medical attendance by prefecture (*n* = 19). Scatter plot comparing the estimated proportion infected based on serological data and the estimated influenza-like illness (ILI) cases with medical attendance based on the notification from sentinel medical institutions. The proportion infected was estimated from the antibody titer during the hemagglutination inhibition assay adopting a cut-off value of 1 : 40.

Figure 5 shows the distribution of the estimated proportion of infections as a function of the estimated ILI cases with medical attendance by prefecture (*n* = 19). There was no indication that a positive correlation exists between the estimated cases with medical attendance and the estimated infections (*r* = −0.177; *P* = 0.47).

## 4. Discussion

The present study analyzed the age profile and geographic pattern of the estimated infections with influenza (H1N1-2009) and ILI cases throughout the course of the first wave of 2009 pandemic. A clear age dependency was seen in all the datasets with the highest estimate among children and the lowest among the elderly. However, the extent of age dependence was different by the dataset, and most importantly, the ratio of estimated ILI cases with medical attendance to estimated infections appeared to decrease as a function of age. With regard to the geographic pattern, we did not observe any positive correlation between the estimated cases with medical attendance and the estimated infections. Namely, the estimated number of ILI cases with attendance based on sentinel surveillance data appeared not to yield appropriate estimates with age and space, perhaps owing to the nonspecific nature of defining influenza-like illness (ILI). In particular, the ILI cases were seriously overestimated among children. Using the HI titer, the estimated infection rates were 28.5% and 23.5% employing cut-off values at 1 : 20 and 1 : 40, respectively, although these should be regarded as slight overestimates due to a small but non negligible coverage of vaccination. To our knowledge, the present study is the first to report the comparative investigation of the magnitude of the first wave for influenza A (H1N1-2009) from Japan. The age profile of seroepidemiological estimates of infection was consistent with the age-profiles reported from Hong Kong and The Netherland [6, 17, 18].

It has been well known that only a portion of ILI cases that are reported through the surveillance system are truly influenza, and those caused by other etiological agents (e.g., other viral infections) contribute to contaminating the number of cases [19, 20]. Thus, there is a possibility that some of the cases reported as ILI were actually attributed to other upper respiratory virus infections such as the respiratory syncytial virus (RSV). Not only the specificity but also the sensitivity of ILI for detecting influenza infections is known not to be high, because there are asymptomatic cases, and moreover, only a fraction of symptomatic cases seek for medical attendance. Experimental studies and household transmission studies agree that approximately 60% of infections with influenza H1N1-2009 would be febrile [21, 22] and only a small portion of the febrile patients may go to the clinic or hospital. Considering the apparent inconsistency between ILI cases and seroepidemiological estimates of infection, we emphasize that the sentinel surveillance data should not be regarded as a data source for estimating the total number of influenza cases, and thus, to measure the burden of influenza based on the notified number of cases with medical attendance. Rather, the sentinel surveillance data should be inspected for other objectives such as the early detection of outbreaks. The primary and secondary objectives of surveillance data including the clinical surveillance and virus isolation data should be systematically reviewed.

 As was widely accepted in other studies during the 2009 pandemic [6, 17, 18], seroepidemiological methods should be sought for directly measuring the morbidity of influenza appropriately, and thus, also for assessing the virulence (e.g., as measured by the infection fatality risk), which is recognized as one of the most important lessons from the 2009 pandemic [23]. Considering the usefulness of seroepidemiological data, it is worth calculating the appropriate sample size of seroepidemiological studies in advance of a seasonal influenza epidemic or a future pandemic [24]. Similarly, as a part of the rapid research response program, one may plan to obtain any clearance of ethical approval in advance of the pandemic event. 

 While mathematical modeling would be useful to understand the underlying epidemiological dynamics of the pandemic, the expected values of the age-specific final size estimates based on early epidemic data only crudely captured the cumulative incidence of infection. Quantitatively, they were not too far from seroepidemiological estimates, and one may regard it as a success of modeling practice based only on the data by the end of containment phase. A plausible reason for underestimating the cumulative incidence among those aged below 12 years is that Japan implemented multiple school closures upon confirmatory diagnosis of at least 1 case across the country during the containment phase. Similarly, a plausible reason for a slight underestimation of the risk among the elderly could be attributed to modeling practice in the early stage of the pandemic during which the transmission was not widespread in the elderly subpopulation. In the future, one should explicitly assess the validity and predictability of the model based on early epidemic data and determine the extent of practical usefulness of modeling results including the use of modeling for real-time forecasting [25–27].

Five limitations should be noted. First, HI antibody assay data were used, but the diagnostic performance, especially the sensitivity, is known to be limited. Second, it should be noted that we have ignored any decay in antibody titer. Third, not only natural infection but also the vaccination contributes to elevating the HI antibody titer, although the vaccination coverage is known to have been very low. We failed to distinguish vaccine-induced seroconversion from natural infections due to shortage of data. The estimated infections in the present study may be, thus, interpreted as the possible “maximum” cumulative incidence of infection. Fourth, there may have been reporting biases by sentinel medical institution (e.g., differential reporting coverage), which we were not able to adjust due to lack of access to the data at sentinel institution levels. Fifth, we have yet to explicitly assess if the nonrandom selection of voluntary participants has introduced any bias.

In summary, we have identified an inconsistency between the cumulative incidence of infections with pandemic influenza H1N1-2009 based on serological data and the cumulative incidence of ILI cases with medical attendance based on sentinel surveillance. It is difficult to appropriately quantify the risk of influenza using the so-called multiplier method, because ILI cases reported from sentinel medical institutions are nonspecific, and an inherent reporting bias is likely introduced. To explicitly estimate the burden of influenza and the virulence of a novel influenza strain, one should plan seroepidemiological study possibly in advance of the epidemic event.
